# Effectiveness of a sensorimotor exercise program on proprioception, balance, muscle strength, functional mobility and risk of falls in older people

**DOI:** 10.3389/fphys.2024.1309161

**Published:** 2024-04-17

**Authors:** Ivelize Freire, Adérito Seixas

**Affiliations:** ^1^ FP-I3ID, FP-BHS, Escola Superior de Saúde Fernando Pessoa, Porto, Portugal; ^2^ LABIOMEP, INEGI-LAETA, Faculdade de Desporto, Universidade do Porto, Porto, Portugal

**Keywords:** balance, elderly, sensorimotor exercises, risk of fall, quality of life

## Abstract

**Introduction:**

Sensory systems provide the necessary information for a motor response to be provided. In this sense, the objective of this study is to evaluate the effectiveness of a sensorimotor exercise program on proprioceptive acuity, balance, muscle strength, functional mobility and risk of falls in institutionalized elderly.

**Methodology:**

56 participants (84.6 ± 8.4 years) were randomly distributed between the control (CG, *n* = 28) and intervention groups (IG, *n* = 28). The CG performed a protocol based on warm-up, muscle strengthening and warm down and the IG performed the same intervention, with the addition of sensorimotor exercises. Joint Position Sensation (JPS) was evaluated in both limbs at angles of 20° and 45°, balance, functional mobility, fear of falling in the elderly and muscle strength of quadriceps, hamstrings, adductors and abductors in both limbs, before and after the 12 weeks of intervention.

**Results:**

Both groups showed gains in muscle strength. When analyzing functionality through Timed Up and Go (TUG), before and after for each group separately, both showed a significant difference (CG *p* = 0.002; IG *p* < 0.001). For the Short Physical Performance Battery (SPPB) variable, there were significant differences in IG in balance (*p* < 0.001), gait speed time (s) (*p* = 0.004) and sit-to-stand (*p* = 0.002). In JPS, significant differences were recorded for Absolute Error 45° Non-Dominant (*p* = 0.045) and Relative Error 45° Non-Dominant (*p* = 0.045) in the CG and Relative Error 45° Non-Dominant for IG (*p* = 0.018). In the Falls Efficacy Scale International (FES-I) variable there were significant improvements in the CG (*p* = 0.006) and in the GI (*p* = 0.002). However, only IG showed significant improvements (*p* = 0.013) for Activities-Specific Balance Confident (ABC) in a comparison between before and after the 12-week research period. When comparing the differences verified with the intervention between CG and IG, only balance SPPB (*p* < 0.001) and sit-to-stand SPPB (*p* = 0.022) showed significant values.

**Conclusion:**

He effectiveness of sensorimotor exercises provides balance gain in the elderly (*p* < 0.001) and positively impacts their confidence (*p* = 0.013) when performing their duties. It is concluded that the protocol presented in its different levels of difficulty is effective and important for the quality of life of the institutionalized sedentary elderly.

## Introduction

Aging has been described as a process, or set of processes, inherent to all living beings and which is expressed by the loss of adaptability and the decrease in functionality (Spirduso, 2005).

Tinetti (2003) demonstrated that approximately 30% of individuals over 65 years of age fall at least once a year and that, in 50% of them, the episode is recurrent, with falls being the main cause of physical damage in the elderly. Half of the cases, that is, 50%, occur due to Tripping due to obstacles that arise during walking is responsible for about 50% of the falls (Berg et al., 1997; Rubenstein and Josephson, 2002).

Conditions related to physiological systems, psychological condition and the use of polypharmacy are considered intrinsic risk factors for falls (Wang and Wollin, 2004; Marin et al., 2007; Sousa L. M. et al., 2017; Kim et al., 2020), as well as lack of physical activity (Basta et al., 2022). The influence of the external environment (objects and furniture placed in risky places, utensils that cause instability, improper lighting or accessibility) and socioeconomic condition are considered extrinsic factors (Lovallo et al., 2010; Chianca et al., 2013; Sousa N. et al., 2017; Kim et al., 2020).

Considering the sensorimotor demands of daily tasks, detecting obstacles, sending information to the nervous system, processing them and choosing the best motor response are skills that the elderly need to constantly develop (Gauchard et al., 2003). For this, the synergistic action of the sensorimotor, vestibular and visual systems, is essential to maintain dynamic stability, static stability, and balance, reducing of the risk of falls in the elderly (Gauchard et al., 2003).

Recent research associates the study of sensorimotor exercises in association with some particular diseases like Parkinson’s, showing positive results in gait and postural control, Fil-Balkan et al. (2018); Calabrò et al. (2019), or Diabetes, with significance results in balance and proprioception. Ahmad et al. (2019); Ahmad et al. (2020). Others, such as Cabo et al. (2023), carried out research with an approach directed at the elderly public without predetermined pathologies. However, the superficial analysis of the results does not take into account the behavior of the variables between the groups, between before and after the intervention and between the differences verified with the intervention. The importance of more research focused on the diseases mentioned must be invested because of the relevance of these pathologies.

In this sense, the objective of this study is to evaluate the effectiveness of a sensorimotor exercise program on proprioceptive acuity, balance, muscular strength, functional mobility and risk of falls in institutionalized elderly people with detailed analysis of the data obtained. In addition to presenting the evolution of exercises in four phases with low production costs. This is an economical and accessible alternative to increase the ability of this sensoriomotor system in elderly.

## Methods

### Study design

A single-blind randomized controlled study was implemented in two Long Stay Institutions in the city of RJ, Brazil.

The study was approved by Plataforma Brasil—National Platform of the Ethics Committee for Research and Human Beings, according to CAAE registration: 27657220.9.0000.8066. All volunteers signed an informed consent form to participate in the study and anonymity and confidentiality were ensured, in agreement with the Declaration of Helsinki. It has been registered at Brazilian Registry of Clinical Trials (ReBEC) by number RBR—6q5299j.

### Participants

Male and female institutionalized elderly from 70 years of age who were under regular medical follow-up, with physical autonomy when leaving bed and who were not performing regular physical activity were included.

Elderly people with physical independence restricted to bed, wheelchair users or who did not have the physical condition to walk, with cognitive impairment, hemodynamic instability, severe heart disease or uncontrolled systemic arterial hypertension verified by the physician were excluded.

Fifty-six participants were recruited and randomly distributed (https://www.randomizer.org/) in two groups: control and intervention.

Over the weeks there were 11 dropouts. Therefore, 21 participants in the intervention group and 24 participants in the control group completed the intervention. The reasons for dropping out were due to lack of interest, aversion to physiotherapy or difficulty adapting to a new routine, despite respect for individual rest, food and medication schedules.

The participants in the control and intervention group were similar at baseline regarding gender, lower limb dominance, fall risk, and age (Table 1).

**TABLE 1:** General characterization of the sample and study groups. Data from nominal variables presented as n (%) and continuous variables presented as mean ± standard deviation.

	Total sample (*n* = 45) (n%)	CG (*n* = 24) n (%)	IG (*n* = 21) n (%)	*p*
Male Participant	12 (26.6)	6 (25)	6 (28.57)	0.073
Female Participant	33 (73.3)	18 (75)	15 (71.42)	
Dominant Right Side	38 (84.4)	21 (87.5)	17 (80.95)	0.689
Dominant Left Side	7 (15.5)	3 (12.5)	4 (19.04)	
Fall risk (FES-I ≥ 31)	26 (57.8)	14 (58.3)	12 (57.1)	0.936
Fall risk (ABC <67%)	27 (60)	14 (58.3)	13 (61.9)	0.807
Age	84.6 ± 8.4	85.13 (8.94)	84.10 ± 7.99	0.688

**FIGURE 1:**
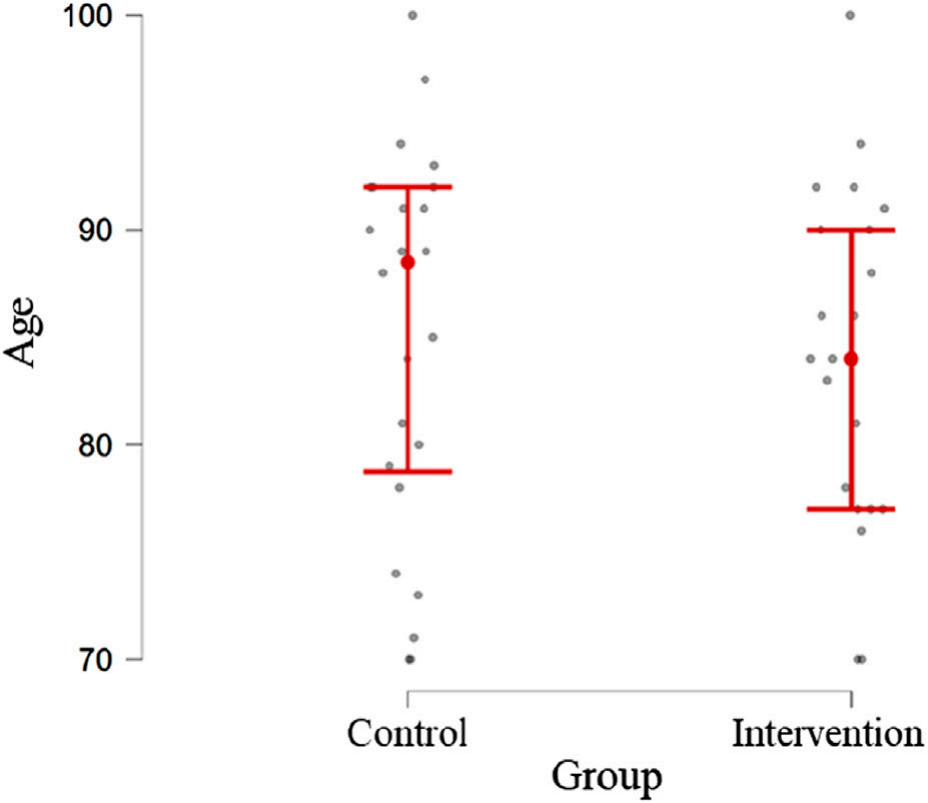
Age distribution of participants in the two groups under study.

It is observed that the majority of participants for both CG and IG are female (75% and 71.42%, respectively) and presented the dominant side as the right [21 participants (87.5%) and 17 participants (80.95%), respectively]. Moreover, in Figure 1, it is possible to analyze the age distribution between the two groups, indicating normality in the distribution of data.

### Statistical analysis

For the statistical analysis of the study data, SPSS software (version 26 for Windows) was used, and a significance level of 5% was considered. After analyzing the distribution of the variables under study, using the ShapiroWilk normality test, it was found that the variables age and variables related to the strength of the participants (muscular strength of the quadriceps, hamstrings, adductors and hip abductors) followed a normal distribution. In this sense, the *t*-test for independent samples was used to compare the average values of these variables between the groups under study, and the *t*-test for paired samples to verify the existence of significant differences between the average values of the variables before and after the intervention. The remaining variables were analyzed using non-parametric tests, specifically the Mann-Whitney test to compare the medians of the variables between the groups under study and the Wilcoxon test to verify the existence of significant differences between the medians of the variables before and after the intervention.

The association between nominal variables was assessed using the Chi-Square test and, whenever necessary, Fisher’s exact test.

### Joint position sense assessment

The assessment of the knee JPS was performed in a sitting position, in an open kinetic chain, with the participant blindfolded, in a silent environment, and active reposition method (Baker et al., 2002; Ribeiro and Oliveira, 2010; Salgado et al., 2015). From the starting positioning (knee flexed at 90°) the participants’ leg was passively moved to extension to one of the test ranges 20° or 45° of knee flexion defined by a goniometer (Baker et al., 2002; Dieling et al., 2014). Participants were instructed to actively sustain the test position for 5s and to return to the starting position. Immediately after, they were instructed to actively reposition the knee to the target position and sustain it for 5 s. Both test ranges in both lower limbs were assessed 3 times and the order of the assessments was randomized (https://www.randomizer.org/).

The knee JPS assessment was recorded using a video camera mounted on a tripod, located at a distance of 2-3 m from the participant. Four markers were fixed to the skin with double-sided hypoallergenic adhesive tape in the following locations: greater trochanter; in the iliotibial tract, level with the posterior crease of the knee when it was flexed at 80°; at the head of the fibula; and in the lateral malleolus (Bennell et al., 2005). Video analysis was carried out in Kinovea Software (version 0.8.15) in which 3 frames from the last 3 s of each positioning/repositioning were analyzed. After calculating the average obtained in the 3 repositioning attempts, 3 errors were calculated: absolute angular error, relative angular error, and variable angular error (Vuolo, 1992). Analyzing the errors, the Microsoft Excel program for Microsoft 365 MSO (Version 2,208 Build 16.0.15601.20148) 64 bits was used.

### Short physical performance battery (SPPB)

When performing the balance test, the participant had to remain for 10 s in each of the following positions: side-by-side, semi-tandem stand and tandem stand and the score was between 0 and 4 points (Guralnik et al., 2000; McDermott et al., 2012).

For the gait speed test, a distance of 4 meters was used, the time in which the participant performed the test was observed and recorded: score 0: test not completed; score 1: time greater than 8.7 s; score 2: time between 6.21 and 8.70 s; score 3: time between 4.82 and 6.20 s and score 4, time less than 4.82 s to complete the test (Guralnik et al., 2000).

The last test to be carried out using the SPPB tool was the 5-time sit-to-stand test, in which five repetitions were performed and the time was recorded, the score was understood between 0–4 (Guralnik et al., 2000).

The total sum of the three tests comprised the final SPPB score comprised between 0 (worst performance) and 12 points (best performance), according to Guralnik et al. (1995), Ferrucci et al. (2000) and Penninx et al. (2000).

A recently carried out study, realized by Welch et al. (2021) presents the usefulness of this tool to be applied among older adults.

### Timed up and go (TUG)

The execution of the TUG test was carried out following the author (Podsiadlo and Richardson, 1991), in which a chair with a seat height of approximately 46 cm was used. The participant was instructed to get up, to cover a distance of 3 meters at a comfortable and safe speed, to turn around and returned to the seat. The time to perform the test was recorded. Before that, the examiner demonstrated the execution of the test and the participant did it experimentally for the first time.

Studies associated with “Frailty syndrome and risk of falling in community-dwelling elderly people,” carried out by Taguchi et al. (2022), used TUG to search for information related to functionality and dynamic balance.

### Falls efficacy scale-international (FES-I)

The instrument includes sixteen statements related to daily life activities and the participants had to indicate their concern about falling when performing them. The scale was applied in the form of an interview. Scores were obtained according to their response: score 1 “not at all concerned,” score 2 “a little concerned,” score 3 “very concerned” and score 4 “extremely concerned” (Camargos et al., 2010).

Zak et al. (2022) showed in their study that the tool FES-I can be an effective assessment tool for addressing the fear of falling issue among older adults. Besides, it can support specialists such as physicians, physiotherapists, psychologists, or psychiatrists in their work.

### Activities-specific balance confidence scale (ABC scale)

The participants indicted their confidence performing sixteen actions, choosing a percentage between 0 (no confidence) and 100 (total confidence). According to Myers et al. (1998), scores above 80% indicate a high level of physical functionality; between 50%–80% represent a moderate level of physical functionality and scores below 50% represent low level of physical functionality.

The choice of this tool can also be supported by current studies, such as that carried out by Elboim-Gabyzon et al. (2019), which states that this scale is a popular, theoretically based, reliable and valid tool designed to assess issues associated with the risk of falls in older people.

### Maximum strength assessment

Strength was assessed with an adapted sphygmomanometer (Bohannon, 1988), which is a common and adequate method to assess muscle strength (Souza et al., 2013). The Modified Sphygmomanometer Test is a promising method because it is low-cost and provides objective measures, according to Souza et al. (2014). The inflatable part was removed, folded into three equal parts and wrapped in a cotton fabric bag. The equipment was previously inflated to 20 mmHg (Perossa et al., 1998). Before measurements, all participants trained the procedure for familiarization.

Dominant and non-dominant quadriceps, hamstring, adductor and abductor were randomly evaluated. When evaluating the quadriceps and hamstring muscle groups, the participant was seated, with their knee and hip joints flexed at 90°, with manual stability from the examiner only in the proximal knee. The adapted sphygmomanometer was positioned in the distal segment of the lower limb for measurement (Bohannon, 1986; Bohannon and Andrews, 1995). On the anterior side, the quadriceps muscles were measured and on the posterior side, the hamstrings.

The participant was positioned in lateral decubitus to measure the abductor muscles. The upper limb in contact with the surface was under the head and the contralateral upper limb in a comfortable position (Perossa et al., 1998). The elderly person was instructed to raise their leg. As for the adductor muscles, the participant in supine position, while the contralateral lower limb was elevated, the adductor muscles of the lower limb to be evaluated tried to cross the midline.

The participant was encouraged through verbal stimuli to perform the maximum force for each muscle three times, lasting 5 s each attempt and resting for 30 s between them (Brown and Weir, 2001). Having performed the arithmetic mean between the values obtained.

The pattern of the script for the application of the instruments followed the following order: firstly, the FES-I and ABC Scale scales were carried out, followed by measuring the maximum muscular strength of the lower limbs with the adapted sphygmomanometer, evaluation of the sense of joint position with the device of the videocamera system and finishing with the SPPB and TUG tests.

The estimated time for using all the instruments described with the participant was 60 min, regardless of the group the participant was in, having seen the similarity of the evaluation methods for both control group and intervention group. The first assessment was carried out before the start of the protocols in both groups.

### Intervention

The study intervention design is summarized in Figure 1. The control group first performed a warm-up corresponding to a 6-min safe walk (Zheng et al., 2013). After that, the protocol was focused on muscle strengthening in which the following exercises were performed (3 sets of 10 repetitions, 2 min rest between sets): 1) patient in dorsal decubitus position with support in the thoracocervical region on the surface, performed the abduction and adduction of the lower limb, with the contralateral lower limb at rest in the position of semiflexion of the knee with unipodal leaning on the bed. The exercise was performed on both limbs. 2) in a sitting position, the patient performed extension (concentric contraction) and flexion (eccentric contraction) of the knee, in an open kinetic chain to strengthen the knee extensor muscles; 3) in a standing position, with the upper limbs on a fixed surface, the participants simultaneously raised both heels from the floor to strengthen the calf muscles; 4) in a standing position, with the upper limbs on a fixed surface, the patient performed unilateral knee flexion (concentric contraction) and extension (eccentric contraction). The estimated initial load, in the elderly participant, was 40% RM with gradual increments until the final load estimated at 80%RM (Rebelo-Marques et al., 2018). Obtaining and modulating the %RM, the Holten Diagram was used, in which, through mathematical calculations, the desired values in %RM were discovered (40%RM, for example), by observing the relationship between the number of repetitions performed by the participant and the percentage that this load represented. Shin guards with multiple values of 0.5 kg were used for this progress (from 40%RM to 80%RM) during the 12-week intervention period.

In the intervention group, all participants performed the same protocol as the control group, with the addition of sensorimotor exercises. The sensorimotor exercises comprised 4 exercises with 4 levels of difficulty:

- “Adhesive Star”: on a soft non-slip Kapazi mat, a star figure made up of up to eight points with adhesive tape, each point measuring up to 40 cm. The participant located in the center of the star would follow the verbal commands by sliding his foot to the requested number. Difficulty levels: i) equal-sized four-pointed star (40 cm); ii) four-pointed star of different sizes (anterior point: 40 cm, posterior point: 30 cm, right point: 40 cm and left point: 30 cm); iii) eight-pointed star of equal sizes (40 cm); iv) eight-pointed star of different sizes (anterior point: 40 cm, posterior point: 30 cm, right point: 40 cm, left point: 30 cm, antero-right diagonal point: 40 cm, postero-right diagonal point: 30 cm, front-left diagonal tip: 30 cm, postero-left diagonal tip: 40 cm).
- “Colored Path”: on a soft non-slip Kapazi mat with 7 Gy paper squares and seven blue paper squares were attached. The squares measuring 15 × 15 cm were arranged in a “zigzag” format. The progressions for this activity were: i) the participant followed the path and came back stepping on the squares of the same color; ii) followed the path of the same color and returned to another color; iii) the participant followed and returned the path alternating colors; iv) the elderly followed and returned the path stepping on the colors to be requested by the researcher at the time of the walk.
- “Rubber Step”: Participant on top of the step to try to balance. The progressions for this exercise were: i) the initial height of the step was 5 cm and the participant threw a light ball with a diameter and circumference of, approximately, 22 cm and 68 cm, respectively, towards the researcher according to her verbal command; ii) the participant had to go up and down the step and after that throw the ball towards the researcher; iii) and iv) respectively repeated commands, with a new step height of 10 cm.
- “Obstacles on the Path”: The participant walked on the Kapazi non-slip soft mat over a 3.6 m route and when faced with obstacles had to perform a triple flexion of the lower limb (dorsiflexion, hip flexion and knee flexion). The evolution for this exercise followed the following order: i) three obstacles with 5 cm in height, 8 cm in width and 40 cm in length placed randomly along the path; ii) three obstacles with new dimensions, each: 10 cm high, 8 cm wide and 40 cm long; iii) presence of three cones (colors: blue, green and white) with a height of 20 cm, each, randomly arranged along the route; iv) mix of obstacles: alternation between all the objects already mentioned.

**FIGURE 2:**
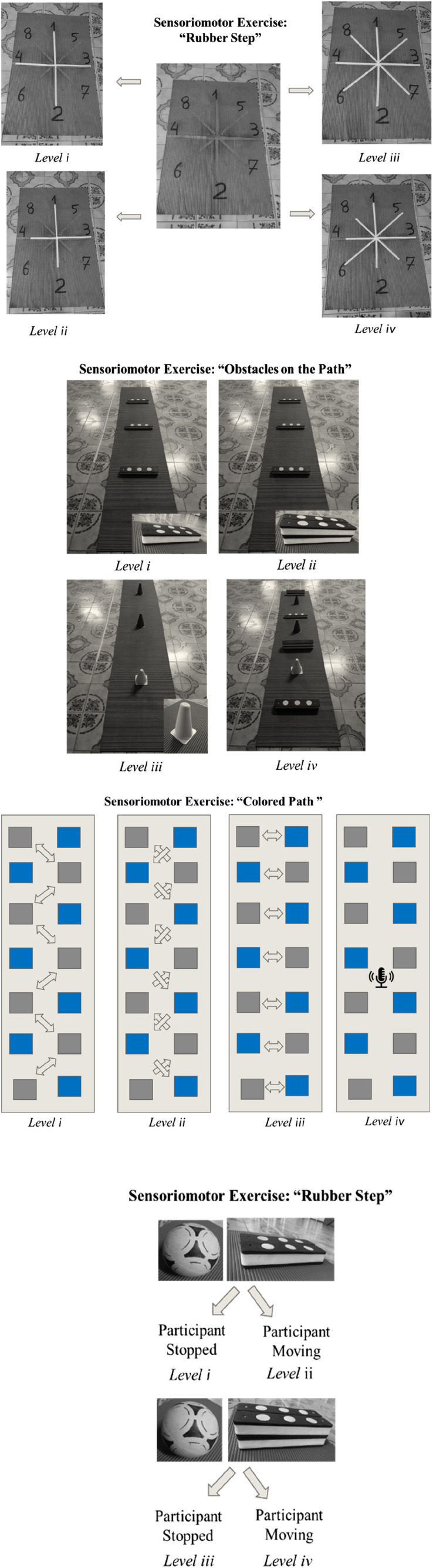
Diagram of steps for each sensorimotor exercise.

Figure 2 shows a summary of the steps of each sensorimotor exercise applied in the study.

The interventions were implemented 3 times/week, according to Rugbeer et al. (2017), during 12 weeks of physiotherapeutic care (Cadore et al., 2013).

**FIGURE 3:**
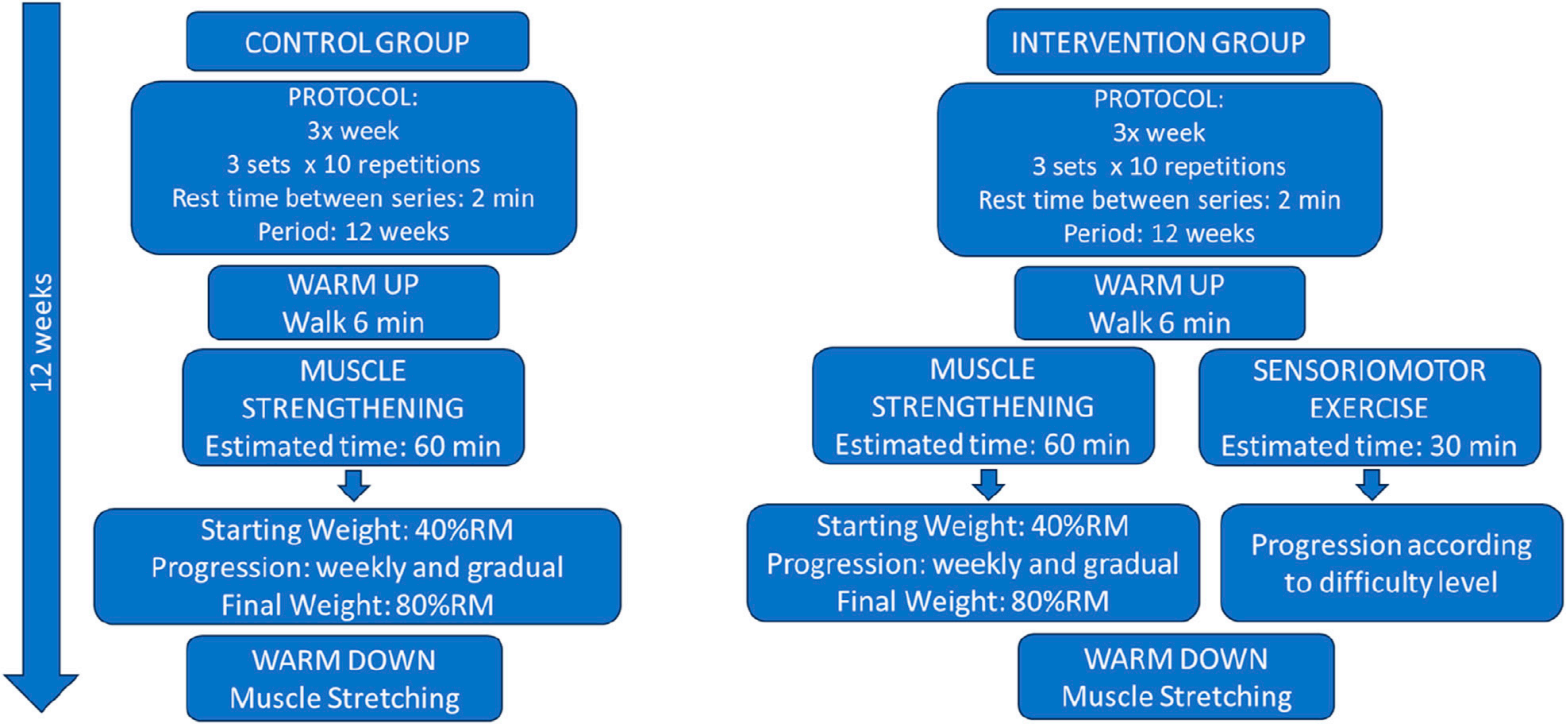
Protocol scheme stipulated for Control and Intervention Groups.

Figure 3 presents the summary of the entire interventional strategy implemented in the study.

The protocols described were based on studies previously carried out and published by Cadore et al. (2013), Bierbaum et al. (2013) and Zheng et al. (2013).

The participant in this intervention group performed the four types of sensorimotor exercises and the progression was according to their level of safety and confidence when performing them. Estimated time for each modality: 6 min. Rest time between modalities: 2 min. Time to perform sensorimotor exercises: 30 min. Total time of the physiotherapeutic session in the intervention group: 90 min.

If any accident, injury or fall occurred to the participant during the research, the local nursing team would be called, as would the responsible doctor, to intervene immediately, through local assessment and care.

If there was a need to travel with the participant to a hospital unit, this would be done to the Institution’s usual unit, providing the researcher with the deserved assistance and monitoring of the case.

If necessary, the Institution’s own emergency protocol should be followed. Considering that the Institution already presented its action measures, it committed to providing the necessary infrastructure to guarantee the safety and wellbeing of everyone involved in the project. The participants read and signed the Letter of Consent. They could give up whenever they wanted. The participant’s family would be informed immediately in the event of any complications, via call notification.

## Results

### Muscle strength

In Table 2, it is possible to note that before intervention the groups were similar in respect to muscle strength. After interventions, significant between-group difference were only noted in the dominant quadriceps muscle strength.

**TABLE 2:** Between-group differences, before and after the intervention for muscle strength (mmHg) [mean ± SD].

	Groups					
	Before			After		
	CG	IG	*p*	CG	IG	*p*
QuadDom	164.98 ± 60.29	186.4 ± 51.04	0.209	206.82 ± 49.69	235.3 ± 44.2	0.05*
QuadnDom	159.57 ± 68.68	178.46 ± 44.2	0.286	199.36 ± 64.24	228.52 ± 43.02	0.085
HAMDom	126.2 ± 40.93	143 ± 39.61	0.171	156.65 ± 47.1	168.94 ± 35.2	0.333
HAMnDom	119.23 ± 49.88	136.96 ± 32.09	0.17	148.63 ± 51.85	168.63 ± 38.95	0.156
AddDom	87.04 ± 16.47	90.06 ± 13.61	0.51	112.55 ± 21.61	113.33 ± 16.89	0.895
AddnDom	82.1 ± 23.73	85.97 ± 14.78	0.523	108.98 ± 26.7	108.36 ± 14.11	0.921
AbdDom	103.17 ± 22.5	100.58 ± 18.19	0.676	136.54 ± 37.03	137.31 ± 23.67	0.933
AbdnDom	98.17 ± 34.18	99.09 ± 19.38	0.914	125.33 ± 34.27	132.07 ± 22.42	0.434

When assessing the within-group differences, it is possible to note in Table 3 that improvements in strength of all assessed muscle groups were verified.

**TABLE 3:** Within-group differences in muscle strength, before and after the intervention, in both groups (mmHg) [mean ± SD].

	Groups					
	CG			IG		
	Before	After	*p*	Before	After	*p*
QuadDom	164.98 ± 60.29	206.82 ± 49.69	<0.001*	186.4 ± 51.04	235.3 ± 44.2	<0.001*
QuadnDom	159.57 ± 68.68	199.36 ± 64.24	0.002*	178.46 ± 44.2	228.52 ± 43.02	<0.001*
HAMDom	126.2 ± 40.93	156.65 ± 47.1	<0.001*	143 ± 39.61	168.94 ± 35.2	<0.001*
HAMnDom	119.23 ± 49.88	148.63 ± 51.85	<0.001*	136.96 ± 32.09	168.63 ± 38.95	<0.001*
AddDom	87.04 ± 16.47	112.55 ± 21.61	<0.001*	90.06 ± 13.61	113.33 ± 16.89	<0.001*
AddnDom	82.1 ± 23.73	108.98 ± 26.7	<0.001*	85.97 ± 14.78	108.36 ± 14.11	<0.001*
AbdDom	103.17 ± 22.5	136.54 ± 37.03	<0.001*	100.58 ± 18.19	137.31 ± 23.67	<0.001*
AbdnDom	98.17 ± 34.18	125.33 ± 34.27	<0.001*	99.09 ± 19.38	132.07 ± 22.42	<0.001*

(*) *p* ≤ 0.05.

Considering that both groups improved in muscle strength, a new variable reflecting the differences in muscle strength (final assessment - initial assessment) was computed for both groups and the results of between-group comparisons is shown in Table 4. No significant differences in muscle strength improvements was found between groups.

**TABLE 4:** Between**-**group comparison in muscle strength improvements (mmHg) [mean ± SD].

	Groups		*p*
	CG	IG	
DifQuadDom	41.83 ± 49.37	48.89 ± 41.68	0.61
DifQuadnDom	39.78 ± 54.11	50.05 ± 40.67	0.481
DifHAMDom	30.44 ± 26.19	25.94 ± 28.09	0.581
DifHAMnDom	29.39 ± 27.33	31.66 ± 24.89	0.773
DifAddDom	25.51 ± 16.18	23.27 ± 14.00	0.624
DifAddnDom	26.87 ± 17.72	22.39 ± 17.77	0.402
DifAbdDom	33.36 ± 29.15	36.72 ± 23.29	0.674
DifAbdnDom	27.15 ± 25.91	32.98 ± 24.94	0.448

### TUG

Similar results were found for the TUG test with absence of significant differences in between-group comparison, in both before and after intervention assessments (Table 5), and statistically significant differences in within group comparison (Table 6) in both groups (CG *p* = 0.002; IG *p* < 0.001). The improvements in TUG test were similar in both groups (Table 7).

**TABLE 5:** Confrontation of groups with each other, before and after the intervention for TUG(s) [median (AIQ)].

	Groups					
	Before			After		
	CG	IG	*p*	CG	IG	*p*
TUG	33.93 (33.47)	32.40 (130.79)	0.785	22.87 (60.80)	22.96 (75.38)	0.873

**TABLE 6:** Related analysis between before and after intervention in both groups for TUG(s) [median (AIQ)].

	Groups					
	CG			IG		
	Before	After	*p*	Before	After	*p*
TUG	33.93 (33.47)	22.87 (60.80)	0.002*	32.40 (130.79)	22.96 (75.38)	<0.001*

(*) *p* ≤ 0.05.

**TABLE 7:** Comparison of the differences verified with the intervention between the two groups for TUG(s) [median (AIQ)].

	Groups		*p*
	CG	IG	
DifTUG	−1.66 (13.71)	−6.31 (80.91)	0.439

### SPPB

The SPPB variable was analyzed in the following aspects: balance, gait speed, gait speed time (s) and sit-to-stand. According to Table 8, balance was significantly different (*p* = 0.003) when compared between groups after 12 weeks, but not before the intervention No significant differences were found for other variables (gait speed, gait speed time (s) and sit-stand).

**TABLE 8:** Statistical results related to the CG and IG groups, before and after intervention for SPPB [median (AIQ)].

	Groups					
	Before			After		
SPPB	CG	IG	*p*	CG	IG	*p*
Balance	1.00 (2.00)	1.00 (1.00)	0.436	1.00 (3.00)	3.00 (1.00)	0.003*
Gait Speed	1.00 (1.00)	1.00 (1.00)	0.557	1.00 (1.00)	1.00 (1.00)	0.956
Gait speed time (s)	14.11 (14.72)	12.48 (16.33)	0.937	10.22 (9.00)	9.09 (8.14)	0.682
Sit-Stand	0.00 (1.00)	0.00 (1.00)	0.925	0.00 (1.00)	1.00 (2.00)	0.149

(*) *p* ≤ 0.05.

Table 9 shows the comparison between the before and after for both groups, CG and IG, showing *p* ≤ 0.05 for balance (*p* < 0.001), gait speed time (s) (*p* = 0.004) and Sit-Stand (*p* = 0.002) to the participants involved in the IG. When analyzing the data difference between the period after the 12 weeks of intervention and the initial period between the two groups, the significance can be proven in the variables DifBal (*p* < 0.001) and DifSitStand (*p* = 0.022), as showed by Table 10.

**TABLE 9:** Analysis of the relationship before and after intervention in both groups for SPPB [median (AIQ)].

	Groups					
	CG			IG		
SPPB	Before	After	*p*	Before	After	*p*
Balance	1.00 (2.00)	1.00 (3.00)	0.317	1.00 (1.00)	3.00 (1.00)	<0.001*
Gait Speed	1.00 (1.00)	1.00 (1.00)	0.739	1.00 (1.00)	1.00 (1.00)	0.129
Gait speed time (s)	14.11 (14.72)	10.22 (9.00)	0.086	12.48 (16.33)	9.09 (8.14)	0.004*
Sit-Stand	0.00 (1.00)	0.00 (1.00)	0.206	0.00 (1.00)	1.00 (2.00)	0.002*

(*) *p* ≤ 0.05.

**TABLE 10:** Statistical analysis of the differences observed with the intervention between the two groups for SPPB [median (AIQ)].

	Groups		*p*
	CG	IG	
DifBalance	0.00 (0.75)	1.00 (1.50)	<0.001*
DifGaitSpeed	0.00 (0.00)	0.00 (0.00)	0.326
DifTGaitSpeed (s)	−1.39 (6.17)	−1.38 (7.75)	0.375
DifSitStand	0.00 (0.00)	1.00 (1.00)	0.022*

(*) *p* ≤ 0.05.

### JPS

With regard to the variable Joint Positions Sense (JPS) it can be observed, in Table 11, that when comparing the groups among themselves, in the circumstances before and after the interventions, only the 20° variable errors showed a significant difference with the dominant limb for after the intervention (*p* = 0.029) and non-dominant limb before the intervention (*p* = 0.007). When comparing the before and after for the groups in particular, it is already possible to verify significant values for the Absolute Error 45° Non-Dom (*p* = 0.045) and Relative Error 45° Non-Dom (*p* = 0.045) in CG and Relative Error 45° Non-Dom for IG (*p* = 0.018), as shown in Table 12. When the differences in periods between CG and IG were analyzed, no variable showed significant values (see Table 13).

**TABLE 11:** Evaluation of difference between themselves, before and after intervention for JPS [median (AIQ)].

	Groups					
	Before			After		
	CG	IG	*p*	CG	IG	*p*
Absolute Error 45° Dom	7.55 (7.49)	7.37 (5.96)	0.474	5.92 (7.39)	4.56 (6.81)	0.082
Absolute Error 45° Não Dom	6.72 (12.12)	7.89 (9.47)	0.413	6.31 (7.63)	7.74 (6.34)	0.211
Relative Error 45° Dom	6.92 (7.61)	7.37 (7.20)	0.625	5.76 (7.55)	4.56 (7.80)	0.265
Relative Error 45° Não Dom	6.72 (12.12)	7.89 (9.64)	0.539	5.20 (8.19)	6.67 (6.67)	0.495
Variable Error 45° Dom	2.34 (2.80)	1.79 (3.36)	0.891	3.31 (3.36)	1.33 (2.15)	0.062
Variable Error 45° Não Dom	3.35 (3.84)	2.19 (0.76)	0.191	1.76 (3.52)	1.64 (2.73)	0.351
Absolute Error 20° Dom	15.17 (13.02)	18.22 (14.37)	0.811	13.98 (19.73)	14.64 (8.80)	0.495
Absolute Error 20° Não Dom	15.36 (14.62)	16.70 (20.67)	0.467	12.61 (12.85)	11.89 (12.80)	0.802
Relative Error 20° Dom	−15.17 (13.02)	−18.22 (14.37)	0.811	−13.98 (19.73)	−14.63 (8.80)	0.495
Relative Error 20° Não Dom	−15.36 (14.62)	−16.70 (20.67)	0.413	−11.89 (12.80)	−11.89 (12.80)	0.767
Variable Error 20° Dom	3.74 (5.31)	4.01 (3.87)	0.82	5.03 (5.40)	3.52 (2.62)	0.029*
Variable Error 20° Não Dom	5.31 (4.68)	2.79 (3.36)	0.007*	4.34 (5.18)	3.89 (2.77)	0.467

(*) *p* ≤ 0.05.

**TABLE 12:** Statistical analysis before and after the intervention in both groups for JPS [median (AIQ)].

	Groups					
	CG			IG		
	Before	After	*p*	Before	After	*p*
Absolute Error 45° Dom	7.55 (7.49)	5.92 (7.39)	0.658	7.37 (5.96)	4.56 (6.81)	0.122
Absolute Error 45° Não Dom	6.72 (12.12)	6.31 (7.63)	0.045*	7.89 (9.47)	7.74 (6.34)	0.187
Relative Error 45° Dom	6.92 (7.61)	5.76 (7.55)	0.989	7.37 (7.20)	4.56 (7.80)	0.217
Relative Error 45° Não Dom	6.72 (12.12)	5.20 (8.19)	0.045*	7.89 (9.64)	6.67 (6.67)	0.018*
Variable Error 45° Dom	2.34 (2.80)	3.31 (3.36)	0.775	1.79 (3.36)	1.33 (2.15)	0.274
Variable Error 45° Não Dom	3.35 (3.84)	1.76 (3.52)	0.073	2.19 (0.76)	1.64 (2.73)	0.808
Absolute Error 20° Dom	15.17 (13.02)	13.98 (19.73)	0.732	18.22 (14.37)	14.64 (8.80)	0.689
Absolute Error 20° Não Dom	15.36 (14.62)	12.61 (12.85)	0.605	16.70 (20.67)	11.89 (12.80)	0.058
Relative Error 20° Dom	−15.17 (13.02)	−13.98 (19.73)	0.71	−18.22 (14.37)	−14.63 (8.80)	0.689
Relative Error 20° Não Dom	−15.36 (14.62)	−11.89 (12.80)	0.503	−16.70 (20.67)	−11.89 (12.80)	0.058
Variable Error 20° Dom	3.74 (5.31)	5.03 (5.40)	0.407	4.01 (3.87)	3.52 (2.62)	0.079
Variable Error 20° Não Dom	5.31 (4.68)	4.34 (5.18)	0.26	2.79 (3.36)	3.89 (2.77)	0.181

(*) *p* ≤ 0.05.

**TABLE 13:** Data related to the comparison of the differences verified with the intervention between the two groups for JPS [median (AIQ)].

	Groups		*p*
	CG	IG	
Dif_AE45D	−0.01 (7.37)	−3.00 (6.76)	0.363
Dif_RE45D	−0.01 (10.24)	−3.15 (7.56)	0.363
Dif_VE45D	0.02 (3.94)	−0.62 (1.90)	0.46
Dif_AE45ND	−2.59 (8.93)	−0.48 (10.19)	0.759
Dif_RE45ND	−2.74 (8.93)	−5.70 (12.32)	0.539
Dif_VE45ND	−1.13 (4.28)	−0.88 (2.88)	0.179
Dif_AE20D	3.11 (19.98)	−1.93 (13.50)	0.649
Dif_RE20D	−3.34 (19.98)	1.93 (13.50)	0.577
Dif_VE20D	1.18 (5.18)	−1.80 (3.47)	0.059
Dif_AE20ND	−0.46 (11.17)	−5.56 (19.79)	0.246
Dif_RE20ND	0.46 (12.78)	5.63 (20.53)	0.339
Dif_VE20ND	−1.68 (6.97)	0.92 (4.64)	0.114

### FES

In Table 14, the FES variable did not show significant importance between the groups for the instant before and after the intervention. However, Table 15 shows *p* = 0.006 for the comparison between before and after for CG and *p* = 0.002 for the same correlation in IG. When this before and after difference (DifFES) are again compared between groups, the value obtained is above *p* ≤ 0.005 (*p* = 0.569), in Table 16.

**TABLE 14:** Contrast of groups among themselves, before and after the intervention for FES [median (AIQ)].

	Groups					
	Before			After		
	CG	IG	*p*	CG	IG	*p*
FES	36.00 (26.00)	35.00 (31.00)	0.927	29.00 (19.00)	27.00 (14.00)	0.855

**TABLE 15:** Comparison results between before and after the intervention in both groups for FES [median (AIQ)].

	Groups					
	CG			IG		
	Before	After	*p*	Before	After	*p*
FES	36.00 (26.00)	29.00 (19.00)	0.006*	35.00 (31.00)	27.00 (14.00)	0.002*

(*) *p* ≤ 0.05.

**TABLE 16:** Analysis of data related to verified differences in the intervention between two groups for FES [median (AIQ)].

	Groups		*p*
	CG	IG	
DifFES	−2.50 (10.50)	−5.00 (15.00)	0.569

### ABC

Tables 17–19 show the existing comparative relationships between the variables associated with the ABC scale. In Table 17, the comparison between the CG and IG for each period, do not show significant differences, regardless of the presence of sensorimotor exercises stipulated in the intervention group.

**TABLE 17:** Statistical understanding between CG e IG, before and after the intervention for ABC [median (AIQ)].

	Groups					
	Before			After		
	CG	IG	*p*	CG	IG	*p*
ABC	51.56 (55.78)	56.87 (42.81)	0.767	73.12 (40.47)	72.50 (28.13)	0.716

**TABLE 18:** Comparison between before and after the intervention in both groups for ABC [median (AIQ)].

	Groups					
	CG			IG		
	Before	After	*p*	Before	After	*p*
ABC	51.56 (55.78)	73.12 (40.47)	0.095	56.87 (42.81)	72.50 (28.13)	0.013*

(*) *p* ≤ 0.05.

**TABLE 19:** Analysis of data related to the ABC difference variable between groups CG and IG [median (AIQ)].

	Groups		*p*
	CG	IG	
DifABC	1.56 (18.75)	5.62 (26.52)	0.356

Only in Table 18 is the significant value evident for the comparison between before and after for IG (*p* = 0.013), however the difference in values between groups in Table 19 did not show significant differences when comparing CG and IG with each other.

## Discussion

The main findings of the present study indicate that a correct interpretation of them, in accordance with previously published trials, can be considered to apply this knowledge to clinical practice.

### Joint position sense

In this study, the intervention programs of both groups do not seem to have caused consistent changes in proprioceptive acuity. However, in the CG there were significant differences in the absolute error at 45° in the non-dominant limb and a significant decrease in the relative error at 45° in the non-dominant limb in both groups. These findings may suggest an increase in proprioceptive acuity at 45° for the non-dominant limb in both groups, which does not seem to be a result of specific sensorimotor exercises, but rather an improvement resulting from the exercise in a global way. In other words, physical activity can be considered as an important factor in maintaining proprioceptive acuity. It is supported by the study carried out by Ribeiro and Oliveira (2010) when referring to the importance of muscle action and muscle spindles as a source of sensory information (from 40° to 60°).

Accordingly, a study published by Liu et al. (2021) states that external stimuli (vibratory movement in the ankle, in the cited article) improved the proprioceptive performance of muscles in the non-dominant limb for a low performance group. The authors argued that the “stochastic resonance” concept, in which the brain manages to improve its responses to weak sensory stimuli as long as there is a certain intensity of noise (provocations that are captured by the sensory system), may have been responsible for the changes.

Additionally, the same authors argue that the existing laterality between the two cerebral hemispheres presents a difference in function and proprioceptive command between the right and left hemispheres depending on whether the limb is dominant or non-dominant. The non-dominant limb would be more related to position control, while the dominant limb would be more related to the movement trajectory, according to research also carried out by Wang and Sainburg (2006). They investigated the transfer pattern between the upper limbs following the adaptation to 30° visuomotor rotations in left and right handers. They concluded, therefore, that there is an asymmetry in the transfer between the dependent limbs related to the cerebral hemisphere. It is likely that in the current study, given the nature of the interventions in the experimental group, support and position control activities (non-dominant limb) were implemented.

On the other hand, Carzoli et al. (2022) reported in their study that nine training sessions for the hip abductors or ankle dorsiflexors in the non-dominant leg in elderly people were carried out. Participants revealed no changes in postural sway before and after any training intervention, as well as in Sit-To-Stand test, Timed Up-and-Go test or maximal voluntary contraction (MVC) torque.

Regarding the fact that the most consistent changes were verified in the evaluation at 45°, it is important to mention that the muscle receptors responsible for proprioceptive acuity in the knee are, according to Olsson et al. (2004), more active between 40° and 60° of flexion (intermediate ranges), while joint receptors are more active close to the range of motion limit (extreme ranges). Considering that strengthening exercises and sensorimotor exercises, due to their nature, may stimulate mainly muscle receptors, which may help understanding the present results.

### Muscle strength

In the present study strength improved in both groups, which was expected considering that both groups included a strengthening program.

Due to the choice of exercise protocol aimed at muscle strengthening in both the control and intervention groups, it was noticed that there was a significant gain in strength in both. Orssatto et al. (2020) states that power training is effective for gaining functional capacity, strength and muscular power in older people.

The data from this study is also in agreement with the systematic review and meta-analysis published by Grgic et al. (2020), in which training using resistance is effective in improving muscle strength in the elderly, including those over 80 years old, in addition to gaining hypertrophy for many participants.

A meta-analysis and systematic review carried out by Carneiro et al. (2021) analyzed elderly people over 65 years of age hospitalized. They carrying out resistance protocols for possible gains in muscular strength, power and functional capacity in contrast to behaviors imposed by the medical team. These are: physiotherapy focused on walking recommended by doctors (Ortiz-Alonso et al., 2020; Sáez de Asteasu et al., 2020; Martínez-Velilla et al., 2021), breathing and stretching exercises (McCullagh et al., 2020), medical support and related professionals (Morton et al., 2007). It can be seen that resistant exercises were considered promising for strength, power and functional capacity in acutely hospitalized elderly people.

Lopez et al. (2018), confirms that despite a great heterogeneity in muscle strengthening protocols aimed at resistive training (frequency, sessions per week, number of repetitions, intensity, number of sets, among others) and their combinations with other multimodal exercises or not, it represents an effective intervention for the weaknesses present in the elderly.

Another study carried out by Amato et al. (2022) in elderly people with Parkinson’s disease when performing resistance training obtained significant results in manual dexterity, static and dynamic balance, reaction time, cervical range of movement, and reduction in bone loss. Considering that more research related to neurodegenerative diseases must be studied and published.

Although muscle strength training was bilateral, in our study, the results show that the dominant quadriceps was the only muscle group that presented significant between-group differences after the 12-week intervention. Considering the absence of significant differences in the improvements in muscle strength in both groups, we are not able to argue that the results in between-group comparisons after the intervention are a consequence of the intervention and further studies are needed to clarify this aspect.

### Falls risk

The intervention group evidenced significant improvements in confidence to practice their daily activities and in the fear of falling. According to Yardley and Smith, (2002) these two conditions (fear of falling and loss of confidence) are important factors for the loss of physical function and social interactions, negatively impacting the elderly’s live.

The study of Sherrington et al. (2019) supports our findings, stating that the choice of exercise related to balance and functional exercises reduces the risk of falls by 24%. Nevertheless when resistance exercises were included, the reduction in the risk of falls improved by 34%. In agreement, Thomas et al. (2019) states that both muscle strength and balance training are effective methods for reducing the risk of falls in the elderly.

**On the other hand,** a study carried out by Wang et al. (2017) states that **only** muscle strengthening training with maximum loads, few repetitions in concentric contraction performed in elderly people, increases the efficiency of type II fibers (increase in size and percentage) for better physical functionality and prevention of falls, even without knowing the impact on muscle morphology.

### Functionality

Regarding functionality, our findings are in agreement with the research of Carvalho et al. (2015) which reported significant differences for the TUG test, with worse results in elderly women with fall history. This is a widely used tool to assess the functional conditions of elderly people as risk of falls (Ortega-Bastidas et al., 2023). However, results for the SPPB test battery were not significantly different between groups.

Study carried out by Vieira de Moraes filho et al. (2020) on individuals with Parkinson’s disease (DP), between 50–80 years old, during 9 weeks, showed a reduction in bradykinesia and improved functional performance in patients with mild to moderate PD when it was applied a progressive resistance training (PRT). Significant time was noted by the group interaction for all functional tests (TUG, thirty-second chair stand test, and Ten Meters Walk Test; all *p* < 0.01) and Bradykinesia subscale (*p* < 0.01).

Moreover, Sousa and Sampaio (2005) reported a significant relationship between muscle strength improvements in lower limbs and the performance in TUG/Functional Reach tests, with the intervention group evidencing improvements in functional activities and prevention of falls, when compared to the control group. Moreover, the authors argue that both resistance and multifactorial training were effective in preventing falls, improving TUG and Functional Reach Test outcomes. Similar results were obtained in studies investigating the effects creative dance (CD) and Tai Chi Chuan as sensory inputs (Zhuang et al., 2014; Joung and Lee, 2019).

In contrast, Gschwind et al. (2015) analyzing video game technology as a sensorimotor stimulus for functional performance but have not reported significant differences resulting from interventions and between the intervention and control groups.

A great variety of exercises targeting the sensorimotor system can be found in the literature. However, clinical and methodological heterogeneity between protocols does not allow to establish a recommended exercise protocol.

### Balance

Some studies on exergames (Nintendo Wii based on biofeedback, for example) have not been effective to increase balance in older adults (Jørgensen, 2014) but reported a high level of motivation and enthusiasm on the part of the participants in carrying out such an intervention. In their study, Torre and Temprado (2022) concluded that despite a high potential to improve cognition and the brain, more systematic studies on the subject are needed. Chu et al. (2022) report that despite being a very promising activity, more robust studies are needed to determine the effects of exergaming on physical health, as well as cognitive and quality of life outcomes. However, Janhunen et al. (2022) and Cikajlo et al. (2020) showed significant results about the importance of exergames for our physical health, included related to balance.

Treml et al. (2013), when analyzing the effectiveness of conventional proprioceptive training in the control group (trampoline, skateboard, board, swing and twist disc) and modified proprioceptive training (adapted and differentiated exercises) associated with the Nintendo Wii in the interventional group, have not reported significant changes in fear of falling (FES-I), and balance in the intervention group. Nevertheless, Ditchburn et al. (2020) support the potential of exergaming to alleviate pain and improve balance in older people with chronic musculoskeletal pain (range and standard deviation of Centre of Pressure -CoP- displacements in the anterior-posterior (AP) and medio-lateral (ML) directions).

In addition, Nascimento et al. (2012) reported that proprioceptive exercises improved balance in the elderly. Also supporting the benefits of proprioceptive exercises in older people are the results of Zheng et al. (2013), evidencing an improvement in balance and proprioception. Thomas et al. (2019) support the idea that balance is a multifactorial quality and should be stimulated by different types of physical activities after analysing eight articles in the quantitative synthesis in their systematic review.

Chittrakul et al. (2020) aimed to determine the effectiveness of a Multi-system Physical Exercise (MPE) for fall prevention and Health-Related Quality of Life in elderly people. The MPE program significantly increased muscle strength and improved proprioception, reaction time, and postural sway to prevent risk of falls. Considering that, according to them, the first cause of falls is usually balance impairment it is crucial to include balance and flexibility training, a resistance exercise strengthening program, and endurance training to improve physical fitness and reducing falls in the community-dwelling elderly (Hosseini et al., 2018; Pillatt et al., 2019).

In the same viewpoint, Donath et al. (2015), states that when muscle strength, resistance, balance and functionality are approached independently, improvements in sensorimotor, neuromuscular and cardiovascular systems are harder to achieve. Therefore, the importance of a multifocal intervention seems to be very promising for improving the quality of life of the elderly, reducing the risks of falls.

Some limitations must be considered when analyzing and interpreting the data. The study was carried out during the COVID-19 pandemic, in which circumstances associated with difficulties in accessing authorized locations for carrying out the research may have had a negative impact. The adaptation of the sphygmomanometer for measuring muscle strength, although commonly used in the literature, had a maximum measurement of 300 mmHg, which has limited strength measurements in few participants. Moreover, sample size, although not small, may have limited the robustness of the study results.

## Conclusion

After presenting and discussing the results, it is possible to conclude that the sensorimotor exercise program in sedentary institutionalized elderly people improved balance (*p* < 0.001), positively impacting the confidence while performing daily activities (*p* = 0.013). Therefore, our findings suggest that sensorimotor exercises should be included in clinical practice. The protocol is easy to apply, low cost for preparing the whole circuit and this study prioritizes a complete program (considering time, frequency, relaxing time and a sequence of exercises. Besides, their respective steps). This will benefit both the applying professional and the elderly person in seeking results.

Additional well-designed studies with more robust samples and follow-up period are required to verify these results and to understand if, after the end of the intervention, the improvements are maintained in time.

## Data Availability

The raw data supporting the conclusion of this article will be made available by the authors, without undue reservation.
